# Tongue brushing enhances the myoelectric activity of the suprahyoid muscles in older adults: a six-week randomized controlled trial

**DOI:** 10.1038/s41598-024-70306-9

**Published:** 2024-08-26

**Authors:** Maya Izumi, Kazuo Sonoki, Sumio Akifusa

**Affiliations:** School of Oral Health Sciences, Faculty of Dentistry, Kyushu Dental University, Kitakyushu, Japan

**Keywords:** Respiratory function, Suprahyoid muscle, Surface electromyography, Tongue brushing, Medical research, Health care, Dentistry, Gerodontics

## Abstract

Tongue brushing improves respiratory function in older adults. Considering connection between the respiratory-related and suprahyoid muscles, this study aimed to investigate whether tongue-brushing interventions can improve myoelectric activity during respiration. A six-week randomized controlled trial was conducted in Kitakyushu, Japan, with 50 participants aged ≥ 65 years. The participants were allocated to the intervention (tongue brushing with routine oral hygiene) or control (routine oral hygiene alone) groups. Surface electromyography (sEMG) was used to assess the myoelectric activity of the suprahyoid muscles during inhalation, exhalation, and forced vital capacity (FVC). A survey was conducted at baseline and the end of the follow-up period. Thirty-six participants were recruited for the analysis. The root mean squares (RMS) of sEMG during exhalation increased significantly at the end of the follow-up period compared with that at baseline in the intervention group [48.7 (18.0–177.5) vs. 64.9 (21.6–163.0), *p* = 0.001], but not in the control group. The generalized linear model revealed that the ratio of change in FVC was correlated with the change in the RMS of sEMG of the suprahyoid muscles during exhalation after adjusting for potential confounders. Tongue brushing enhances the myoelectric activity of the suprahyoid muscle.

## Introduction

According to a statistical report from Japan, approximately 70% of patients with pneumonia are older adults aged 75 years or older, and more than 70% of cases of pneumonia occurring in adults aged 70 years or older are aspiration pneumonia^1^. Sarcopenic dysphagia, a multifaceted condition that causes swallowing difficulty due to a reduction in the mass and strength of the muscles used in swallowing, is associated with aging^2^. Presbyphagia refers to the alterations in the swallowing process typically observed in older individuals, and it is often linked to a reduction in the strength of the tongue muscles and the upward movement of the hyoid bone during swallowing^3,4^. The suprahyoid muscles, which comprise the mylohyoid, digastric, stylohyoid, and geniohyoid muscles, forms the floor of the oral cavity. Its functions include lowering the mandible, and elevating the larynx during swallowing. Thus, dysfunction or impairment of the suprahyoid muscles is closely associated with swallowing difficulties as it plays a key role in elevating the larynx and facilitating the pharyngeal phase of swallowing^5–8^. In patients with swallowing dysfunction caused by weakness of the suprahyoid muscles, insufficient elevation of the hyoid bone and larynx can lead to the inadequate opening of the upper esophageal sphincter, resulting in swallowing difficulties, such as residue in the pharynx and aspiration, in the pharyngeal phase^9,10^. A previous study demonstrated that a reduction in the movement of the hyoid bone and larynx during swallowing below the first-quartile boundaries increases the risk of aspiration and post-swallow residues^11^. Jaw opening exercises^12^, tongue strengthening exercises^13,14^, Shaker exercise^15,16^, and the Mendelsohn maneuver^17^ can be used to train the suprahyoid muscle. Strengthening of the respiratory muscles has been employed in swallowing training as it improves coughing strength and strengthens the suprahyoid muscles as secondary muscles^18–20^, suggesting that the suprahyoid muscles anatomically connect the respiratory muscles through the endothoracic fascia^21^. Previous studies have demonstrated that tongue brushing enhances respiratory function in older adults^22,23^. Since the suprahyoid muscles are related to the tongue and respiratory muscles, we hypothesized that tongue brushing may improve respiratory function by strengthening the suprahyoid muscles. When measuring myoelectricity, methods such as intramuscular electromyography (EMG)^24^, microelectrode EMG^25^, ultrasonographic EMG^26^, magnetomyography^27^, and surface EMG (sEMG)^20^ are considered. Intramuscular and microelectrode EMG are precise but invasive methods. Ultrasonographic EMG and magnetomyography are non-invasive but provide indirect measurements of electric activity. In comparison, sEMG measures muscle electric activity non-invasively, can track multiple muscles at once, and offers real-time feedback. Therefore, this study aimed to investigate the potential impact of tongue brushing on the suprahyoid muscles using sEMG.

## Methods

### Study setting and population

This randomized controlled trial was conducted at two facilities for outpatient rehabilitation in Kitakyushu City, Fukuoka Prefecture, Japan, between August 2022 and March 2023. Fifty individuals aged 65 years or older were invited to participate in this study. As eligibility criteria, individuals who were unable to communicate verbally or maintain a sitting position were excluded from the study, as these criteria are necessary for the accurate assessment of respiratory and swallowing functions. All participants were non-smokers and received a normal oral diet. The participants did not receive any other interventions related to oral function rehabilitation during the follow-up period. This study was approved by the Institutional Review Board for Clinical Research of the Kyushu Dental University (No. 21-51). In accordance with the ethical guidelines, all participants or their legal guardians were informed of the purpose of the study and provided informed consent prior to data collection. The study is registered in the “Effect of oral care on improving coughing ability to prevent aspiration pneumonitis” trial registry under the registration number UMIN0000 33696. The date of first trial registration of the study is 09/08/2018.

### Study design

**Figure 1:**
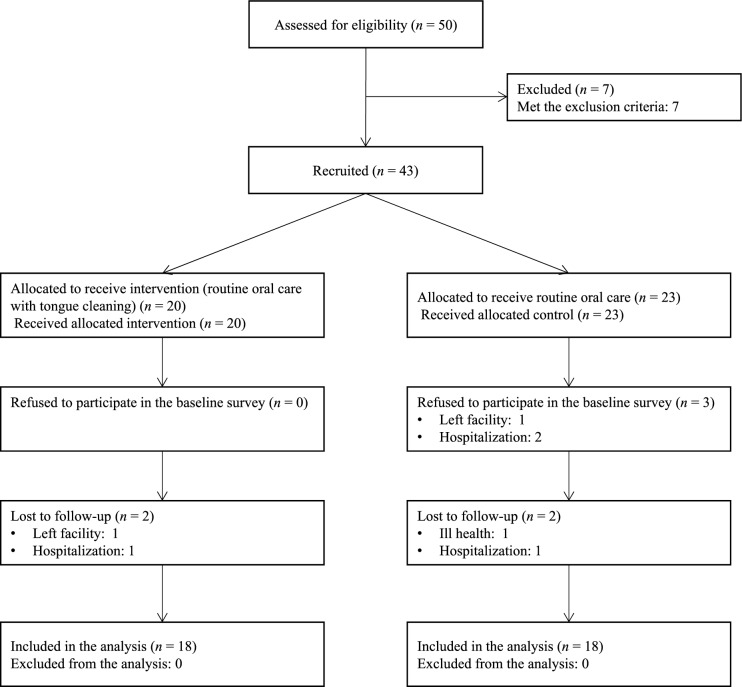
Flow diagram of the study population.

This study was conducted in accordance with the CONSORT statement for randomized trials of non-pharmacological treatments^28^. Figure 1 summarizes the parallel trial design. Among the 50 individuals assessed for eligibility, seven were excluded as they were unable to communicate verbally or maintain a sitting position. Thus, 43 participants were randomly assigned to the intervention (routine oral care with tongue brushing; n = 20; allocation ratio: 46.5%) or control (routine oral care alone; n = 23; allocation ratio: 53.5%) groups using random sampling numbers after the baseline assessments. M.I. generated the random allocation sequence, enrolled participants, and assigned participants to interventions. However, three participants in the control group were excluded from the baseline assessments due to the following reasons: one participant left the facility and two participants were hospitalized. The examiners placed the sEMG electrodes and conducting the outcome assessments and the participants were blinded to the group allocation.

The participants allocated to the control group performed standard oral care by themselves after each meal, using a toothbrush and an interdental brush during the six-week follow-up period. In addition to regular oral care, the participants in the intervention group performed tongue brushing using a mucosal brush (DENT. ERAC 510; Lion Dental Products, Tokyo, Japan). A standard tongue-brushing method was employed, which involved gently brushing the tongue from the base to the apex for 10 strokes, to maintain consistency in the intervention. Each tongue brushing session lasted for approximately 10 s. The bristles of the brush were bent by applying adequate pressure during each stroke^22,23^. The participants were instructed to clean their tongues twice daily: once in the morning and once in the evening after meals, following an instructional video provided by a trained dental hygienist. The instructions were as follows: when brushing your tongue, do so without toothpaste, using a brush moistened with water. Open your mouth wide, extend your tongue forward, and slightly tilt your head down to avoid triggering the gag reflex. Brush from the base of the tongue towards the tip, applying enough pressure to bend the bristles, approximately 150–200 g, and repeat this process 10 times. During the follow-up period, one participant in the intervention group and one participant in the control group were hospitalized, and one participant from the intervention group left the facility. Additionally, one participant from the control group did not participate in the 6-week follow-up survey due to ill health. Therefore, 36 participants (intervention group, *n* = 18; control group, *n* = 18) were included in the analysis. There were no reports of the harmful or unintended effects of tongue brushing in either group.

### Intervention period

Our previous study demonstrated that four weeks of tongue brushing intervention effectively improved expiratory function, as assessed by peak expiratory flow^22^. Considering that the suprahyoid muscles are smaller than the respiratory muscle group and may not significantly benefit from tongue brushing in terms of functional improvement, we extended the intervention period by an additional two weeks.

### Data collection

The following variables were evaluated at the beginning of the study: tongue hygiene, the status of the remaining teeth, swallowing function, activities of daily living (ADL), cognitive function, underlying diseases, nutritional status, tongue pressure, and respiratory function. In addition, tongue hygiene, swallowing function, respiratory function, and tongue pressure were assessed at the end of the follow-up period. Data regarding demographic characteristics, physical health status, and cognitive function were obtained through a standard questionnaire and medical records.

### Oral health status and swallowing function

Oral examinations were performed by a trained dentist. The degree of occlusal support was determined by assessing the posterior tooth contacts, excluding the third molars, and the total number of functional tooth units (FTUs)^29^. The maximum achievable score for FTU was 12. The tongue coating index (TCI), a measure that assesses the amount of coating present on the dorsum of the tongue through visual inspection, was used to assess tongue hygiene^30^. The dorsum of the tongue was divided into nine sections, and each area was rated on a scale of 0–2, and the mean value was calculated from these ratings. The swallowing function was evaluated using the modified water swallow test (MWST), with a reported sensitivity of 70% and specificity of 88% in predicting aspiration^31^. The assessment entailed administering 3 mL of cold water into the participant’s mouth using a 10-mL syringe, followed by instructing the participant to swallow. Subsequently, each participant was assigned a score ranging from 1 to 5, with each score indicating the following: 1, an inability to swallow with choking and/or breathing difficulties; 2, successful swallowing but with changes in breathing; 3, successful swallowing without changes in breathing but with choking and/or wet hoarseness; 4, successful swallowing without choking or wet hoarseness; and 5, successful swallowing without choking or wet hoarseness with the ability to perform an additional dry swallow within 30 s. Participants with scores of 4 or 5 were instructed to swallow their saliva twice within 30 s. The test was repeated twice, and the lower score was used as the final score.

### ADL, cognitive activity, nutrition status, and comorbidities

The functional dependence level of the participants in basic activities of daily living (ADL) was evaluated using the Barthel Index (BI), which encompasses the domains of personal care and mobility. The BI scoring system consists of scores ranging from 0 to 100, with higher scores indicating a greater degree of independence in performing basic ADLs^32^. The Mini-Mental State Examination (MMSE), which includes the domains of registration, orientation, attention, concentration, memory, language, and the ability to follow simple commands, was used to evaluate cognitive function^33^. The scores of MMSE ranged from 0 to 30, with higher scores indicating better cognitive function. The nutritional status was assessed using the Mini Nutritional Assessment Short Form (MNA-SF). The scores of MNA-SF ranged from 0 to 14, with higher scores indicating a better nutritional state^34^.

### Maximum tongue pressure and respiratory function

A tongue pressure measurement device (JMS Co., TPM-01, Tokyo, Japan) was used to evaluate the tongue pressure^35^. The measurements were performed thrice, and the maximum value was recorded as the tongue pressure. A simple electronic spirometer (CHESTGRAPH HI-105; CHEST M. I., Tokyo, Japan) was used to measure the forced vital capacity (FVC) and peak expiratory flow rate (PEFR)^36^. All measurements were performed by a trained dentist.

### Surface electromyography data collection

**Figure 2:**
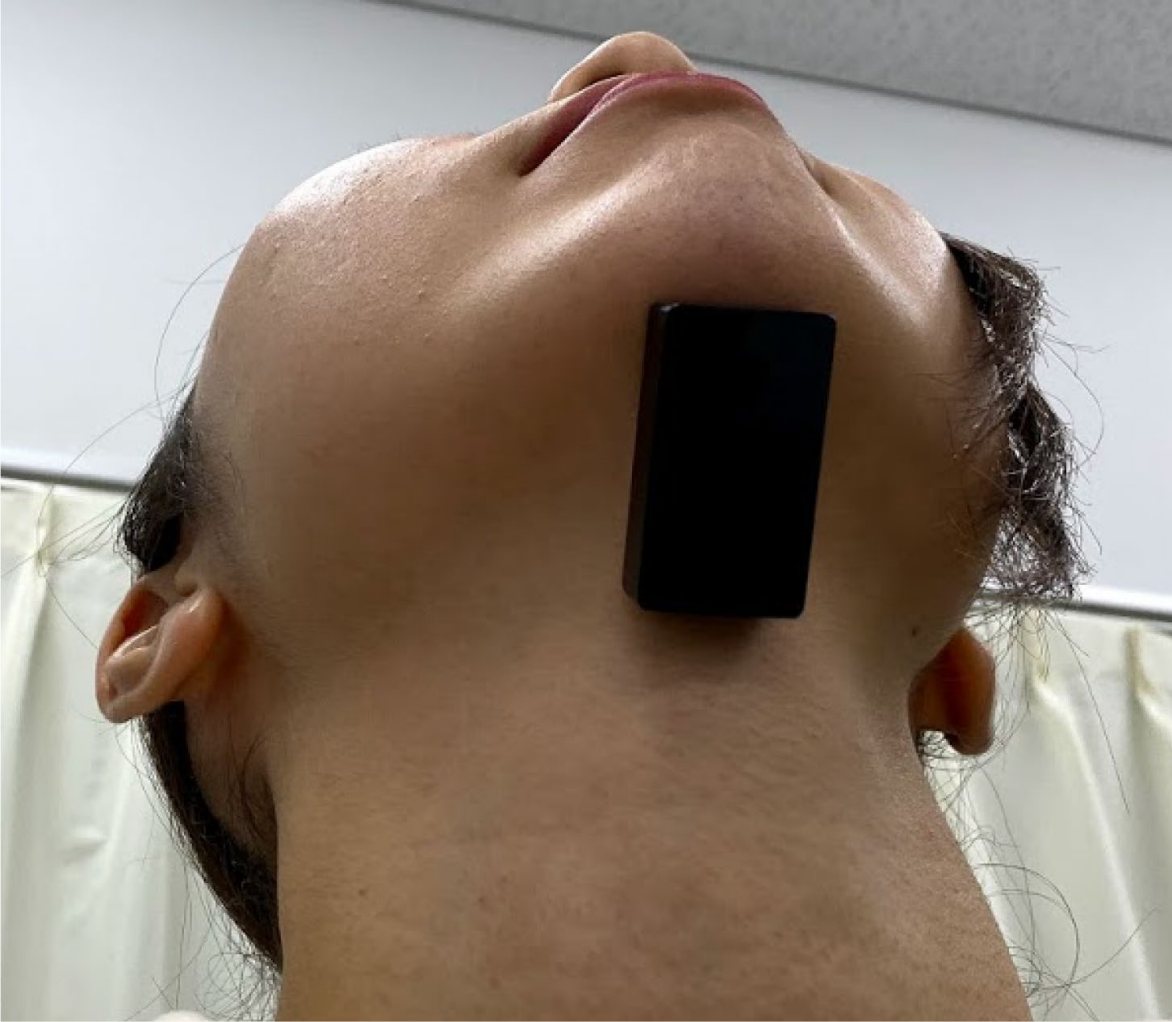
Attachment region of electrode.

Surface electromyography (sEMG) was used to evaluate the activity of the suprahyoid muscles. A wireless sEMG sensor device equipped with three bipolar Ag/AgCl electrodes spaced 10 mm apart (Logical Product Co., Ltd., Fukuoka, Japan) and dry-type electrodes were used in the assessment. Prior to electrode attachment, hair was removed as necessary, and the skin was pre-treated using Nuprep gel® (NIHON SANTEKU Co., Ltd., Osaka, Japan), which contains abrasive particles, and alcohol cotton to decrease the contact resistance between the electrode and the skin. A small quantity of Electrode Gel (Signagel®, Parker Laboratories Inc., NJ) was applied to the electrode to improve electrical conductivity. Subsequently, the electrode was placed parallel to the orientation of the muscle fibers between the mental protuberance and hyoid bone (Fig. 2), and the placement was confirmed by palpation and evaluating the muscle contraction while uttering an “A” sound. A wired data logger (LP-WS1311, Logical Product Co., Ltd.) was used to record the synchronized signal. Additionally, we employed an elastic bandage over the electrodes to further minimize any movement and ensure stable recordings during muscle activity. The signals were digitized using LabVIEW Ver.7.8.11 (Logical Product Co., Ltd.), and the sEMG sampling rate was set at 1000 Hz for analog-to-digital conversion. Bandpass and notch filters at 19.6–442 Hz and 60 Hz, respectively, were applied to eliminate signal noise. The sEMG data were presented as microvolt root mean square (RMS) after full-wave rectification using Map1038 (NIHON SANTEKU Co., Ltd.). The myoelectricity of the suprahyoid muscles was measured while performing various actions, such as inhalation, exhalation, tongue pressure, swallowing 3 mL of water, and tongue-brushing performed by a dentist. Experienced dental hygienist performed the evaluations in a noise-free room, and follow-up measurements were obtained after the intervention to determine the changes in muscle strength. We confirmed the repeatability by having healthy subjects perform the Shaker exercise, which is known to elevate the activity potential of the suprahyoid muscles. The sEMG measurements consistently detected this increased activity, ensuring the protocol’s repeatability and reliability.

### Outcome

The primary outcome was the difference between the RMS of sEMG of the suprahyoid muscles at baseline and the end of the follow-up in the intervention and control groups. The secondary outcome was the difference in respiratory function between baseline and the end of the follow-up period in the intervention and control groups.

### Sample size

The G*Power 3 software program^37^ was used to determine a sample size of 35 for a two-tailed test with an alpha error rate of 0.05, a power of 0.8, and an effect size of 0.6.

### Statistical analysis

Descriptive statistics were used to describe the participant characteristics. Since the data were not normally distributed, all values are presented as medians (minimum − maximum). The differences between the groups were evaluated using the Mann–Whitney *U* test. Within-group differences were assessed using Wilcoxon signed-rank tests for continuous variables. The chi-squared test was used to compare the differences in categorical variables. Multiple comparisons were performed using the Kruskal–Wallis test, followed by the Steel test as a post-hoc test. Spearman’s rank correlation coefficient was used to evaluate the correlation between two variables. A generalized linear model adjusted for sex, age, and the number of teeth was used to evaluate the correlation between the ratio of change in FVC and the ratio of the change in the RMS of sEMG of the suprahyoid muscles during exhalation. All analyses were performed using SPSS statistical software version 22 (SPSS, Chicago, IL, USA). Statistical significance was set at *p* < 0.05.

## Results

### Study participants and baseline characteristics

Thirty-six participants, comprising 18 participants each in the intervention and control groups, were included in the analysis. None of the study participants had general muscle disorders, neuromuscular disorders, or disorders affecting neuromuscular performance. The median age of the participants was 88 (62–96) and 88 (68–94) years in the intervention and control groups, respectively. Table 1 presents the characteristics of the participants in both groups. With the exception of the number of teeth, no statistically significant differences were observed in either group in terms of age, sex ratio, body mass index, cognitive function, ADL, nutritional status, comorbidity status, posterior occlusion status, or caries experience. Comparison of the respiratory function, tongue pressure, and swallowing function at baseline and at the end of the follow-up period in both groups revealed that the respiratory function assessed using FVC [1.3 (0.2–2.6) L to 1.5 (0.4–3.3) L, *p* = 0.007] and PEF [1.1 (0.4–3.9) L/s to 1.4 (0.5–4.6) L/s, *p* = 0.035] increased significantly in the intervention group but not in the control group (Table 2). Tongue pressure and swallowing function, assessed using MWST, did not change in either group. There were no important harms or unintended effects in each group.

**Table 1:** Characteristics of the participants in the intervention and control groups.

Variables	Intervention group (*n* = 18)	Control group (*n* = 18)	*p*
Age (*m*)	88 (62–96)	88 (68–94)	0.723^a^
Sex (*n*)			
Man	6 (33.3%)	3 (16.7%)	0.248^b^
Woman	12 (66.7%)	15 (83.3%)	
BMI (*m*, kg/m^2^)	20.8 (15.6–30.3)	20.9 (16.4–37.4)	0.733^a^
MMSE (*m*)	27.5 (20–29)	26 (0–30)	0.867^a^
MNA (*m*)	12 (8–14)	12 (9–14)	0.960^a^
Barthel index (*m*)	92.5 (30–100)	90 (5–100)	0.246^a^
Number of teeth (*m*)	24 (0–30)	7 (0–28)	0.020^a^
Total FTU (*m*)	10.5 (0–12)	12 (0–12)	0.851^a^
DMFT (*m*)	21 (8–28)	26 (0–28)	0.577^a^
CCI (*m*)	1 (0–3)	1 (0–5)	0.468^a^
FVC (*m*, L)	1.3 (0.2–2.6)	1.5 (0.7–2.3)	0.351^a^
PEF (*m*, L/s)	1.1 (0.4–3.9)	1.1 (0.5–5)	0.527^a^
Tongue pressure (*m*, kPa)	21.1 (0–36.6)	26.3 (6.7–35.8)	0.261^a^
MWST (*m*)	5 (3–5)	5 (3–5)	0.349^a^
sEMG (RMS)			
Inhalation	43.3 (8.3–171.5)	34.0 (14.9–123.1)	0.658^a^
Exhalation	48.7 (18–177.5)	49.5 (19.4–200.3)	0.874^a^
Tongue push	57.0 (16–247.7)	52.7 (20.3–123.0)	0.255^a^
Swallowing	42.4 (18–167.9)	33.3 (19.5–63.6)	0.467^a^
Tongue cleaning	98.2 (41–329.5)	98.2 (25.1–277.2)	0.658^a^

^a^: Mann–Whitney *U* test, ^b^: chi square test. Continuous variables are presented as median (minimum–maximum).

BMI, body mass index; MMSE, mini-mental state examination; MNA, mini nutritional assessment; FTU, functional tooth unit; DMFT, decayed, missing, and filling teeth; CCI, Charlson comorbidity index; FVC: forced vital capacity; PEF, peak expiratory rate, MWST, modified water swallowing test; sEMG, surface electromyography; RMS, root mean square.

**Table 2:** Comparison between the variables at baseline and six weeks in intervention or control group.

Variables	Intervention group			Control group			*p*^‡^
	Baseline	6-weeks later	*p*-value^†^	Baseline	6-weeks later	*p*-value^†^	
FVC (*m*, L)	1.3 (0.2–2.6)	1.5 (0.4–3.3)	0.007	1.5 (0.7–2.3)	1.2 (0.4–2.5)	0.133	0.409
PEFR (*m*, L/s)	1.1 (0.4–3.9)	1.4 (0.5–4.6)	0.035	1.1 (0.5–5)	1.1 (0.5–4.2)	0.396	0.186
Tongue pressure (*m*, kPa)	21.1 (0–36.6)	20.4 (12.6–34.4)	0.472	26.3 (6.7–35.8)	24.9 (6.9–35.2)	0.861	0.331
MWST (*m*)	5 (3–5)	5 (3–5)	0.317	5 (3–5)	5 (2–5)	0.916	0.719
sEMG (*m*, RSM)							
Inhalation	43.3 (8.3–171.5)**	42.0 (14.2–84.2)**	0.184	34 (14.9–123.1)**	34.0 (13.6–123.1)**	0.050	0.019
Exhalation	48.7 (18.0–177.5)**	64.9 (21.6–163.0)**	0.001	49.5 (19.4–200.3)**	46.0 (19.4–200.3)*	0.060	< 0.001
Tongue pressure	57.0 (16.0–247.7)*	68.0 (16.0–291.6)	0.022	52.7 (20.3–123.0)*	44.7 (19.5–123.0)**	0.101	0.028
Swallowing	42.4 (18.0–167.9)**	47.0 (26.9–224.5)**	0.007	33.3 (19.5–63.6)**	32.3 (19.2–63.6)**	0.386	0.016
Tongue cleaning	98.2 (41.0–329.5)	135.7 (50.1–260.9)	0.043	98.2 (25.1–277.2)	87.8 (25.1–277.2)	0.084	0.015
*p*-value^⁋^	< 0.001	< 0.001		< 0.001	< 0.001		

^†^: The variables at baseline and 6-weels later are compared using the Wilcoxon signed-rank sum test.

^‡^: The intervention and control groups are compared using the Mann–Whitney *U* test 6-weeks from baseline.

^⁋^: RSMs of sEMG are compared using the Kruskal–Wallis test.

*: *p* < 0.05 and **: *p* < 0.01 when comparing to tongue cleaning, analysed using Steel test as post hoc test for Kruskal–Wallis test.

FVC, forced vital capacity; PEFR, peak expiratory flow rate; MWST, modified water swallowing test; sEMG, surface electromyography; RMS, root mean square.

### Effect of tongue brushing on suprahyoid muscles

Regarding the myoelectricity of the suprahyoid muscle, the RMSs of the sEMG increased significantly in the intervention group while performing all actions except inhalation (Table 2). However, the myoelectric activity of the suprahyoid muscles remained unchanged in the control group. Comparison between the myoelectricity readings of the intervention and control groups obtained six weeks later revealed that the myoelectricities for all actions were significantly higher in the intervention group than those in the control group. Notably, compared with those of other activities, the myoelectric activity while performing tongue brushing was significantly higher regardless of the group or follow-up period, and the values were 1.4–3.0 times higher. The calculated effect sizes for the Wilcoxon signed-rank test and Mann–Whitney *U* test were 0.492 and 0.985, respectively.

Table 3 presents the comparison of the ratio of the changes in myoelectricity while performing various actions to the baseline myoelectricity level. All ratios showed a statistically significant increase in the intervention group except during inhalation; however, no changes were observed in the control group.

**Table 3:** Comparison of the ratio of change in myoelectricity between baseline and six weeks later.

Action	Intervention group	*p*-value	Control group	*p*-value
Inhalation	1.1 (0.7–2.7)	0.133	1.0 (0.7–1.2)	0.075
Exhalation	1.2 (0.8–2.4)	0.001	1.0 (0.8–1.3)	0.084
Tongue pressure	1.2 (0.8–3.6)	0.031	1.0 (0.4–1.1)	0.087
Swallowing	1.3 (0.8–5.1)	0.007	1.0 (0.7–1.4)	0.386
Tongue cleaning	1.1 (0.9–2.0)	0.020	1.0 (0.6–1.1)	0.071

The data were standardized based on each individual’s ‘before’ measurements. The ‘after’ data were evaluated in proportion to these ‘before’ measurements, ensuring a more accurate comparison of muscle potential changes. The ‘before’ measurements (e.g., 1 as reference) and ‘after’ measurements are compared using the Mann–Whitney *U* test in each group.

### Analysis using a generalized linear model

The correlation between the ratio of change in FVC, PEFR, and the RMS of sEMG of the suprahyoid muscles during exhalation was analyzed to investigate whether a correlation was present between the changes in respiratory function and myoelectric activity of the suprahyoid muscles based on the tongue-brushing intervention. Table 4 presents the correlation efficiencies of the variables. The ratio of change in FVC (r = 0.442, *p* = 0.007), but not in PEFR, was significantly correlated with the ratio of change in the RMS of sEMG. Therefore, the correlation between the ratio of change in FVC and the RMS of sEMG of the suprahyoid muscles during exhalation was analyzed using a generalized linear model. The results of the model showed that the ratio of change in FVC was positively correlated with the ratio of change in the RMS of sEMG of the suprahyoid muscles (B ± SE = 0.4 ± 0.4, *p* = 0.003; Table 5).

**Table 4:** Correlation between the ratio of change in FVC, PEFR, and sEMG (RMS) of the suprahyoid muscles during exhalation.

		Ratio of change	
		FVC	PEFR
Ratio of change in sEMG (RMS) of suprahyoid muscles during exhalation	Correlation efficiency	0.442	0.251
	*p*-value	0.007	0.140

FVC, forced vital capacity; PEFR, peak expiratory flow rate; sEMG, surface electromyography; RMS, root mean square.

**Table 5:** Analysis for ratio of change in FVC by the generalized linear model.

Variables	B ± SE	*p*
Ration of change in sEMG (RMS) of suprahyoid muscles during exhalation	0.4 ± 0.4	0.003
Age	0 ± 0	0.868
Sex	0.3 ± − 0.8	0.493
Number of teeth	0 ± 0	0.377

FVC, forced vital capacity; sEMG, surface electromyography; RMS, root mean square; B, partial regression coefficient; SE, standard error.

## Discussion

The present study evaluated the myoelectric activity of the suprahyoid muscles during various actions, including forced inhalation and exhalation for respiratory function, the force of the tongue pushing against the hard palate for tongue function, and swallowing water for swallowing function. These myoelectric activities, assessed using the RMS of sEMG, increased significantly after performing tongue brushing twice daily for six weeks. The myoelectric activity while performing tongue brushing was significantly higher than that measured while performing other actions. Thus, our findings suggest that tongue brushing may be effective in enhancing the activity of the suprahyoid muscles, similar respiratory muscle training^18–20^ or tongue strengthening exercises^13,14^. An inherent advantage of tongue brushing, when compared with other methods for the training of the suprahyoid muscles, is that it can be performed by individuals with a passive status. In addition, since tongue brushing can be performed easily by non-dental health workers or non-professional caregivers, it can be applied to individuals with cognitive impairment or lower ADL on a daily basis.

The mechanism by which tongue brushing enhances the activity of the suprahyoid muscles remains unknown. Given that activity in these muscles increased during tongue brushing in the intervention group (Tables 2 and 3), a direct effect on their activation is suggested. A recent experimental study with rats showed that tongue exercises enlarged their lingual cortical motor area, indicating the possibility of motor cortex plasticity induced by tongue training^38^. Further research is needed to explore whether tongue brushing influences this process through the cranial nerve system. Considering that the suprahyoid muscles are anatomically linked to the respiratory muscles via the endothoracic fascia, the mechanical action of tongue brushing stimulates the suprahyoid muscles and respiratory muscles involved in the tongue brushing process, which can lead to increased muscle strength and coordination over time^21^. In addition, tongue brushing can improve sensory feedback from the oral cavity, leading to better coordination and control of the suprahyoid muscles during breathing and swallowing activities^21^. These mechanisms may collectively contribute to the observed improvements in suprahyoid muscle activity and respiratory function in individuals who engage in regular tongue brushing.

Tongue brushing intervention improved the respiratory function of the participants in the present study, similar to previous study^22,23^. The facility staff assisted with tongue brushing in the previous studies; in contrast, the participants performed tongue brushing in the current study. Nevertheless, similar to the results reported by a previous study, tongue brushing was effective in improving respiratory function. Thus, self-tongue brushing is an effective intervention.

Myoelectric activity during inhalation was not improved by tongue-brushing intervention. The diaphragm and respiratory accessory muscles, such as the intercostal, pectoralis minor, sternocleidomastoid, scalene, and other upper limb muscles, are used during inhalation^39^. The diaphragm is the main muscle used during respiration, and the chest cavity is expanded by flattening the diaphragm and lowering the ribs, which enables air to be drawn into the lungs. The accessory respiratory muscles work in conjunction with the diaphragm to expand the chest and draw air into the lungs^40^. The muscles required for inhalation may vary from person to person. However, the suprahyoid muscles may help open the larynx during inhalation if an individual is unable to open the larynx sufficiently^41^. While the exact reasons are unclear, the function of the suprahyoid muscle group during inspiration is primarily auxiliary; hence, it may not have changed significantly after the intervention.

Despite an increase in myoelectric activity during tongue pressure, the actual tongue pressure did not improve with tongue brushing interventions, which is inconsistent with the findings of previous studies^22,23^. This inconsistency may be attributed to the differences in participant characteristics, as the participants in the previous studies were residents of nursing homes with lower ADL and cognitive function, whereas those in the present study were receiving outpatient rehabilitation. The reason underlying the limited effect of tongue brushing on the force required to push the tongue toward the palate remains unclear. Although the suprahyoid muscles are involved in pushing the tongue toward the palate, the main muscles responsible for this action may be the intrinsic muscles of the tongue. Therefore, tongue brushing may have a limited effect on the intrinsic muscles of the tongue. Further investigation is warranted to understand the impact of tongue brushing on the intrinsic muscles of the tongue.

The present study has several limitations. First, the sample size was relatively small, and dropouts during the follow-up period were unavoidable given the age of the participants. Additionally, this study did not account for gender differences due to the small sample size. Second, we did not conduct a physiological analysis of the intrinsic muscles of the tongue in this study. The effectiveness of the intervention could be influenced by tongue tension during brushing. Future research could explore the relationship between tongue brushing and the myogenic potential of the intrinsic muscles of the tongue to provide a more comprehensive understanding of the intervention’s mechanisms. Third, the number of teeth in the control group was significantly lower than that in the intervention group. A previous study demonstrated that swallowing dysfunction^42^ or aspiration-related onset of fever^43^ is associated with the occlusion of the posterior teeth, not with the number of remaining teeth, suggesting that the number of remaining teeth may not be correlated with the effect of tongue brushing on myoelectric activity. As an additional analysis, a generalized linear model was used to evaluate the RMSs of sEMG at the end of the follow-up period (Supplemental Table 1). The analysis revealed that each RMS of sEMG at the end of the follow-up period was dependent on the intervention and RMS at baseline but not on the number of remaining teeth. Forth, limitation of using sEMG is that it measures muscle activity from the skin surface, which makes it difficult to evaluate the activity of deep muscles. Additionally, sEMG can pick up signals from adjacent muscles, potentially interfering with the accurate measurement of specific muscle activities. Despite these limitations, sEMG is widely used in research due to its convenience and ability to provide real-time feedback. Fifth, we did not consider the use of medications or the disorders that can affect muscle activity in our exclusion criteria. The use of such medications or the disorders could potentially influence the results of the myoelectrical measurements. Future studies should account for this factor to ensure a more comprehensive understanding of the effects on muscle activity.

Future research will focus on understanding the long-term effects of tongue brushing on suprahyoid muscle activity and respiratory function through larger, longitudinal studies. Investigating the benefits of tongue brushing in populations with respiratory or neuromuscular conditions will also be a priority. These studies will provide a deeper understanding and broader applicability of our findings.

## Conclusions

Tongue brushing performed for a duration of six weeks enhanced the myoelectric activity of the suprahyoid muscles, as demonstrated by the sEMG findings. Myoelectricity measured while performing tongue brushing was significantly higher than that during exhalation, tongue pressure, and swallowing. In addition, the ratio of change in FVC was correlated with the change in the RMS of sEMG activity in the suprahyoid muscles during exhalation. Thus, tongue brushing can effectively enhance the activity of the suprahyoid muscles in older adults.

### Supplementary Information

Supplementary Table 1.

## Data Availability

The anonymized data collected are available as open data via the ZENODO online data repository: https://zenodo.org/records/10065210.
