# Supplementary material for: Tongue brushing enhances the myoelectric activity of the suprahyoid muscles in older adults: a six-week randomized controlled trial
Source: Sci Rep. 2024 Aug 26;14:19746. doi: 10.1038/s41598-024-70306-9 (PMC11347627; doi:10.1038/s41598-024-70306-9)
Supplement: Supplementary file 1 — Supplementary Table 1. [file 41598_2024_70306_MOESM1_ESM.docx]

**Supplemental table 1.** Analysis for RMS during various action at end of follow up period by generalized linear model

Dependent variable: RMS during tongue cleaning at end of follow up period

| Variables | B ± SE | p-value |
| --- | --- | --- |
| Intervention (reference: control) | 29.1 ± 7.8 | <0.001 |
| Sex (reference: woman) | 0.9 ± 8.7 | 0.917 |
| Age | 0.8 ± 0.5 | 0.122 |
| Number of teeth | -0.3 ± 0.4 | 0.444 |
| RMS at baseline | 0.9 ± 0.1 | <0.001 |

Dependent variable: RMS during swallowing at end of follow up period

| Variables | B ± SE | p-value |
| --- | --- | --- |
| Intervention (reference: control) | 31.8 ± 10.4 | 0.002 |
| Sex (reference: woman) | -13.0 ± 11.0 | 0.240 |
| Age | 0.1 ± 0.7 | 0.844 |
| Number of teeth | -0.8 ± 0.5 | 0.116 |
| RMS at baseline | 0.8 ± 0.2 | <0.001 |

Dependent variable: RMS during tongue pressure at end of follow up period

| Variables | B ± SE | p-value |
| --- | --- | --- |
| Intervention (reference: control) | 22.9 ± 10.1 | 0.024 |
| Sex (reference: woman) | 2.5 ± 11.2 | 0.822 |
| Age | 0.4 ± 0.7 | 0.555 |
| Number of teeth | 0.9 ± 0.5 | 0.062 |
| RMS at baseline | 1.0 ± 0.1 | <0.001 |

Dependent variable: RMS during exhalation at end of follow up period

| Variables | B ± SE | p-value |
| --- | --- | --- |
| Intervention (reference: control) | 19.4 ± 4.4 | <0.001 |
| Sex (reference: woman) | 3.7 ± 5.0 | 0.456 |
| Age | 0.4 ± 0.3 | 0.214 |
| Number of teeth | 0 ± 0.2 | 0.812 |
| RMS at baseline | 1.0 ± 0.1 | <0.001 |

B, partial regression coefficient; SE, standard error; RMS: root mean square.
